# Rho GTPase-Activating Protein 35 rs1052667 Polymorphism and Osteosarcoma Risk and Prognosis

**DOI:** 10.1155/2014/396947

**Published:** 2014-07-20

**Authors:** Jinmin Zhao, Hua Xu, Maolin He, Zhe Wang, Yang Wu

**Affiliations:** ^1^Department of Orthopaedics Trauma and Hand Surgery, The First Affiliated Hospital of Guangxi Medical University, Nanning, Guangxi Zhuang Autonomous Region 530021, China; ^2^Research Center for Regenerative Medicine, Guangxi Medical University, Nanning, Guangxi Zhuang Autonomous Region 530021, China; ^3^Center for Education Evaluation & Faculty Development, Guangxi Medical University, Nanning, Guangxi Zhuang Autonomous Region 530021, China; ^4^Division of Spinal Surgery, The First Affiliated Hospital of Guangxi Medical University, Nanning, Guangxi Zhuang Autonomous Region 530021, China

## Abstract

The Rho GTPase-activating protein 35 (ARHGAP35), an important Rho family GTPase-activating protein, may be associated with tumorigenesis of some tumors. Here, we investigated the relationship between an important polymorphic variant at 3′-UTR of this gene (rs1052667) and osteosarcoma risk and prognosis. This hospital-based case-control study, including 247 osteosarcoma patients and 428 age-, sex-, and race-matched healthy controls, was conducted in Guangxi population. Genotypes were tested using TaqMan PCR technique. We found a significant difference in the frequency of rs1052667 genotypes between cases and controls. Compared with the homozygote of rs1052667 C alleles (rs1052667-CC), the genotypes with rs1052667 T alleles (namely, rs1052667-CT or -TT) increased osteosarcoma risk (odds ratios: 2.41 and 7.35, resp.). Moreover, rs1052667 polymorphism was correlated with such pathological features of osteosarcoma as tumor size, tumor grade, and tumor metastasis. Additionally, this polymorphism also modified the overall survival and recurrence-free survival of osteosarcoma cases. Like tumor grade, ARHGAP35 rs1052667 polymorphism was an independent prognostic factor influencing the survival of osteosarcoma. These results suggest that ARHGAP35 rs1052667 polymorphism may be associated with osteosarcoma risk and prognosis.

## 1. Instruction

Osteosarcoma is the most frequent primary malignant bone tumor and usually occurs in patients between 10 and 25 years of age [1, 2]. In the past several years, the 5-year survival of patients with osteosarcoma has significantly improved because of the combined treatment (neoadjuvant chemotherapy, surgery, and adjuvant chemotherapy) [2, 3]. However, about 80% of patients would eventually develop metastatic disease following surgical treatment, and outcome remains poor for these patients [2–4].

Therefore, a better understanding of its basic biology is urgently needed to identify its risk and prognostic markers. Several studies have reported potential associations of common genetic variants with osteosarcoma risk in biologically plausible pathways. This suggests that the genetic factors could play important roles in the pathogenesis of this malignant tumor [5, 6].

The Rho GTPase-activating protein 35 (ARHGAP35, also called GRLF1 and p190RhoGAP) is an important Rho family GTPase-activating protein, and is identified as a tyrosine-phosphorylated protein associated with p120RasGAP in v-Src transformed cells [7–14]. Functionally, it mainly plays a crucial role in regulating cytoskeletal rearrangements, cell spreading and migration, and endothelial barrier function [15–18]. Recent data have shown that this protein can regulate cell proliferation and the dysregulation of ARHGAP35 may be associated with gliomas and breast cancer [19–21]. A common polymorphism at 3′-untranslated region (3′-UTR) of this gene, namely, rs1052667 C > T, has been identified. However, it is unclear whether this polymorphism correlates with tumor. Therefore, we specifically conducted a hospital-based case-control study to examine whether ARHGAP35 rs1052667 polymorphism modifies osteosarcoma risk and prognosis.

## 2. Materials and Methods

### 2.1. Study Population

The present study was approved by the ethics committees of the hospitals involved in this study. This hospital-based case-control study was conducted in Guangxi Zhuang Autonomous Region, China, a relatively high incident area of osteosarcoma. All osteosarcoma patients and control individuals were residents of Guangxi Zhuang Autonomous Region and were recruited from the affiliated hospitals of Guangxi Medical University between January 1996 and August 2005. All cases were histopathologically confirmed. During the same period, control subjects without a history of cancer were randomly selected from a pool of healthy volunteers who visited the general health check-up center of the same hospitals because of their routine scheduled physical exams [22, 23]. To control the effects of confounders, the controls were individually matched (1 : 1 or 2 : 1) to cases based on ethnicity (Han, Minority), sex, and age (±5 years). In this study, a total of 247 cases and 428 controls, representing 97% of eligible cases and 92% of eligible controls, were enrolled, interviewed, and included in the final analysis. After giving written consent, demographic information and clinical pathological data (including age, sex, race, smoking and drinking status, disease history, tumor history, tumor size, tumor grade, and tumor site, etc.) were collected using a standard interviewer administered questionnaire and/or medical records. At the same time, 2 mL of peripheral blood was obtained for analyzing the genotypes of ARHGAP35 rs1052667 polymorphism. All subjects did not have chemotherapy or radiotherapy history before samples collection.

Among 247 osteosarcoma cases, about 57 percent (140/247) of osteosarcomas located in femur. All cases received surgical resection of primary tumor; however, only fifty-seven cases underwent the radical treatment (including both curative resection and adjuvant chemotherapy). In this study, tumor grade was evaluated according to Broders' grading system [24]. Low grade was defined as tumor type with well or moderately differentiated tumor cells (less than 50% undifferentiated cells), whereas high grade was defined as tumor type with poorly or anaplastic/pleomorphic differentiated tumor cells (more than 50% undifferentiated cells) [25].

### 2.2. DNA Detraction

Leukocytes were isolated from peripheral venous blood samples from all tumor patients and control subjects by standard procedures. DNA was then extracted from leukocyte samples by standard phenol-chloroform extraction and ethanol precipitation. DNA samples were stored at −20°C until additional analysis.

### 2.3. Genotyping

The ARHGAP35 rs1052667 genotypes were analyzed by TaqMan polymerase chain reaction (PCR) on an iCycler iQ real-time PCR detection system (iQ5, Bio-Rad, Hercules, CA, USA). The corresponding TaqMan SNP Genotyping Assay Kit (cat# 4351379) was obtained from Applied Biosystems, Carlsbad, CA, USA. TaqMan PCR was performed in total volume of 25 *µ*L consisting of 1 × TaqMAN Universal Master Mix II (cat# 4440041, Applied Biosystems), 1 × TaqMan SNP Genotyping Assay Mix (including both primers and probes, cat# C_16007053_10), and about 75 ng of genomic DNA. Cycling conditions were 95°C for 30 s, and 50 cycles of 95°C for 15 s, and 60°C for 1 min. For quality control, laboratory personnel were blinded to case and control status. Controls were included in each run, and repeated genotyping and sequencing of a random 20% subset yielded 100% identical genotypes.

### 2.4. Osteosarcoma Patients Follow-Up

For survival analysis, we followed all osteosarcoma cases. All patients underwent serial monitoring every 2 months for the first 2 years and semiannually thereafter for detection of any recurrence. In this study, the last follow-up day was December 31, 2013, and survival status was confirmed by clinic records and either patient or family contact. The duration of overall survival (OS) was defined as from the date of curative treatment to the date of death or last known date alive, whereas the recurrence-free survival (RFS) was defined as from the date of curative treatment to the date of tumor recurrence or last known date alive.

### 2.5. Statistical Analysis

All analyses were performed with the statistical package for social science (SPSS) version 18 (SPSS Institute, Chicago, IL, USA). Pearson's *χ* ^2^ test or Fisher's exact test was used to test the differences between osteosarcoma patients and control subjects in the distribution of gender, age, race, and ARHGAP35 rs1052667 genotypes. Because this study was based on an individually matched design, conditional logistic regression was used to evaluate odds ratios (ORs) and 95% confidence intervals (CIs) for risk of osteosarcoma. Kaplan-Meier survival analysis (with the log-rank test) was used to elucidate the relationship between ARHGAP35 rs1052667 polymorphism and osteosarcoma prognosis. Hazard ratios (HRs) and 95% CIs for ARHGAP35 genotypes were calculated from a multivariate Cox regression model (with stepwise forward selection based on the likelihood ratio test). In the present study, a *P*  value of < 0.05 was considered statistically significant.

## 3. Results

### 3.1. Demographic and Clinic Characteristics of the Subjects

In this study, 247 osteosarcoma cases and 428 controls were included in the final analysis. The demographic characteristics of all cases and controls are shown in Table 1. The mean age, gender ratio, smoking and drinking status, and race distribution are of the same levels in both control and osteosarcoma groups (*P* > 0.05).

### 3.2. ARHGAP35 Polymorphism Increased Osteosarcoma Risk

Table 2 summarized the genotypic and allelic distribution of ARHGAP35 rs1052667 polymorphism for both tumor patients and controls. Genotype frequent distribution in controls fitted the Hardy-Weinberg equilibrium well. The heterozygous genotype with rs1052667 C and T allele (rs1052667-CT) and the variant homozygous genotype with rs1052667 T allele (rs1052667-TT) were more frequent among cases than among the controls (*P* < 0.01), resulting in an Ser allele frequency of 32.0% in cases and 11.9% in controls. Logistic regression analysis exhibited that the adjusted OR for osteosarcoma for these individuals carrying rs1052667-CT compared with those exhibiting the homozygote for C alleles (rs1052667-CC) was 2.41 (95% CI, 1.64–3.55) and the corresponding OR for those featuring rs1052667-TT was 7.35 (95% CI, 3.95–13.68). These results showed that osteosarcoma risk was associated with the number of rs1052667 T alleles.

### 3.3. ARHGAP35 Polymorphism and Osteosarcoma Risk Stratified by Gender, Age, and Race

To evaluate possible interactive effects of matching factors (including gender, age, and race) and ARHGAP35 rs1052667 polymorphism on osteosarcoma risk, we performed a series of bivariate stratified analyses by matching factors (Table 3). Because of the small number of subjects with rs1052667-TT among different strata, genotypes rs1052667-CT and rs1052667-TT were combined into one stratum (also called rs1052667-CT/TT). Similar risk values for osteosarcoma were found among Han subjects and among minority participants (adjusted ORs were 3.40 and 3.39, resp.). Similar results were also found in the stratified analysis between rs1052667 polymorphism and other two matching variables. Likelihood ratio tests for interaction of the stratified variables and ARHGAP35 genotypes showed that these matching factors did not modulate the effects of this polymorphism on osteosarcoma risk (*P*_interaction_ > 0.05; Table 3). This suggested that these factors should be effectually manipulated and should not modify the association between this polymorphism and osteosarcoma risk.

### 3.4. ARHGAP35 Polymorphism Modified Osteosarcoma Prognosis

**Figure 1:**
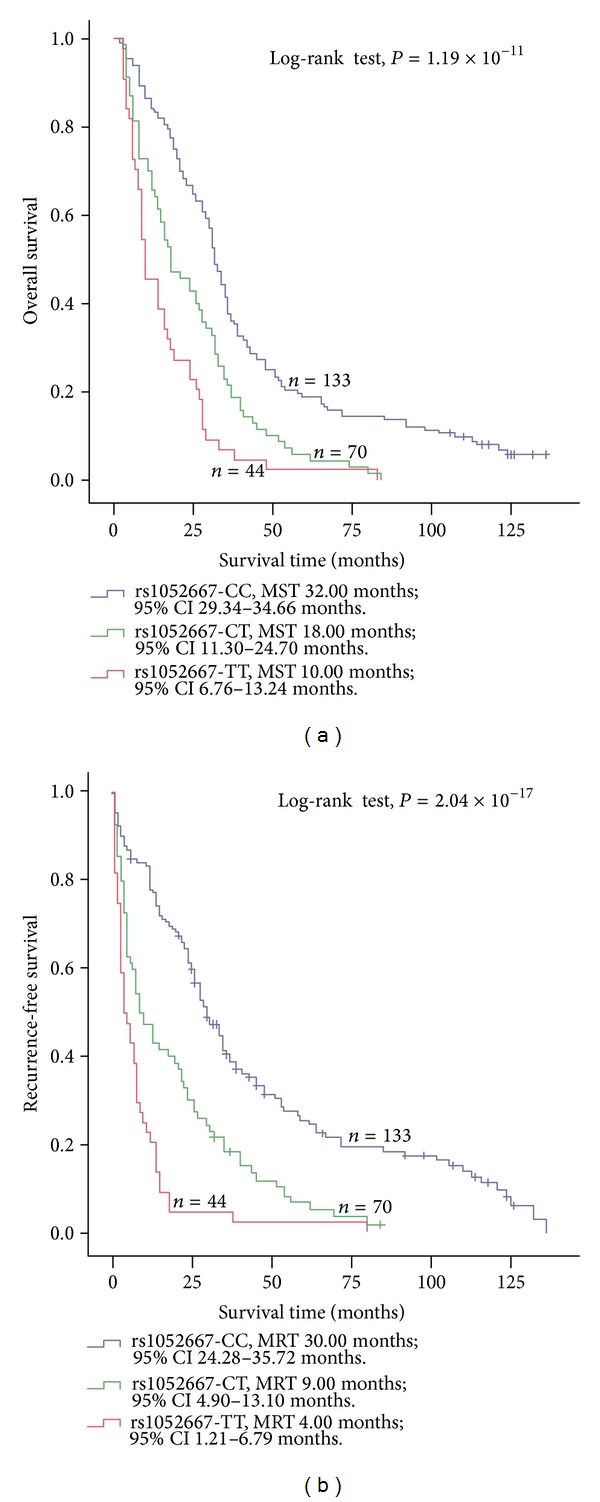
Association between ARHGAP35 rs1052667 polymorphism and osteosarcoma prognosis in 247 osteosarcoma patients. ARHGAP35 rs1052667 polymorphism was correlated with (a) the overall survival and (b) the recurrence-free survival of osteosarcoma. Cumulative hazard function was plotted by the Kaplan-Meier methodology and the *P* value was calculated with two-sided log-rank tests. MST, the median overall survival time; MRT, the median tumor recurrence-free survival time.

**Figure 2:**
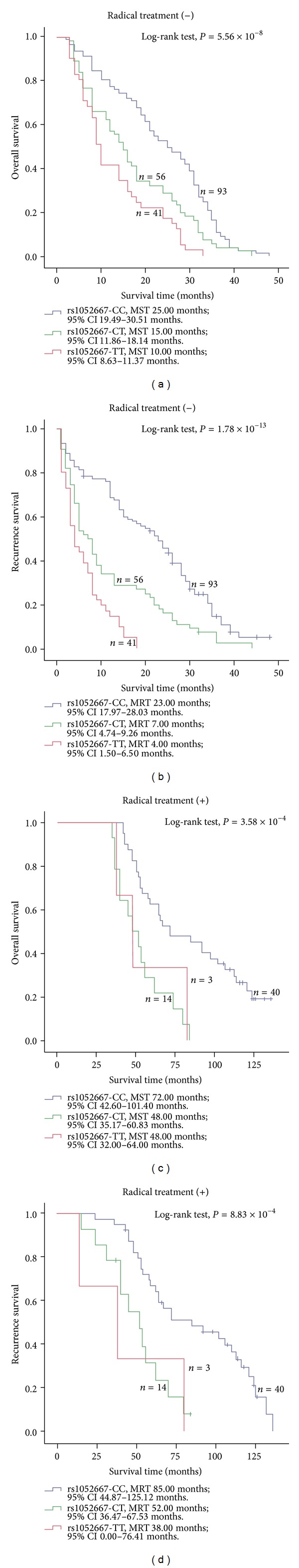
Survival analysis of ARHGAP35 rs1052667 polymorphism in strata of treatment status. According to whether cases received radical treatment (RT) including both surgical resection and adjuvant chemotherapy, 247 osteosarcoma cases were divided into two groups: RT (+) and RT (−). ((a), (c)) Overall survival and ARHGAP35 rs1052667 polymorphism in strata of RT status. ((b), (d)) Tumor recurrence-free survival and ARHGAP35 rs1052667 polymorphism in strata of status. Cumulative hazard function was plotted by Kaplan-Meier's methodology, and *P* value was calculated with two-sided log-rank tests. MST, the median overall survival time; MRT, the median tumor recurrence-free survival time.

To investigate the effects of ARHGAP35 polymorphism on outcome of osteosarcoma patients, we followed all cases and analyzed the survival information of all osteosarcoma cases. During the follow-up period of these patients, 222 faced tumor recurrence with 15.9% of the 5-year RFS rate, and 238 died with 12.5% of the five-year OS rate. Kaplan-Meier survival analysis showed that patients with ARHGAP35 rs1052667 T alleles featured a significantly poorer prognosis than those with rs1052667-CC (*P* is 1.19 × 10^−11^ for OS and *P* is 2.04 × 10^−17^ for RFS, resp.; Figures 1(a) and 1(b)). Considering that some patients did not accomplish entire adjuvant chemotherapy because of poor economic conditions, we stratified the analysis of the correlation between ARHGAP35 genotypes and osteosarcoma outcome by the radical treatment status to explore whether this difference affected the results (Figure 2). Among these cases receiving the radical treatment (Figures 2(c) and 2(d)), shorter median overall survival time (MST) and shorter median tumor recurrence-free survival time (MRT) were found in cases having risk genotypes (including ARHGAP35 rs1052667-CT and -TT) than in those without risk genotypes. Similar results were observed in the nonradical treatment stratum (Figures 2(a), and 2(b)). Multivariate cox regression analysis (with stepwise forward selection based on likelihood ratio test) was next performed to determine whether ARHGAP35 rs1052667 polymorphism was an independent predictor of osteosarcoma cases. The results exhibited that the genotypes with rs1052667 T alleles increased the dying risk of tumor patients compared with rs1052667-CC (HRs: 1.57 for rs1052667-CT and 1.91 for rs1052667-TT, resp.). Risk role was also found in the RFS analysis; the corresponding HRs were 1.82 for rs1052667-CT and 2.53 for rs1052667-TT, respectively (Table 4). Taken together, these results implied that this polymorphism could be used as an independent prognostic marker for osteosarcoma.

### 3.5. ARHGAP35 Polymorphism Correlated with the Clinic-Pathological Features of Osteosarcoma Patients

To explore whether ARHGAP35 rs1052667 polymorphism correlated with the clinical pathological features of osteosarcoma, an association analysis of the risk genotypes (rs1052667-CT/TT) or the nonrisk genotype (rs1052667-CC) and the clinical pathological characteristics of osteosarcoma was performed separately. Results showed that these osteosarcoma cases with risk genotypes of ARHGAP35, compared to those without risk genotypes, faced larger tumor size (OR is 4.85), lower tumor differentiation (OR is 4.07), and higher metastasis risk (OR is 2.78; Table 5). However, this polymorphism did not affect other features.

## 4. Discussion

To the best of our knowledge, no studies have investigated the role of ARHGAP35 rs1052667 polymorphism in the risk of osteosarcoma. In this study, we analyzed the association between aforementioned polymorphism and the risk of osteosarcoma among Guangxi population and found ARHGAP35 rs1052667 T alleles increased osteosarcoma risk (adjusted OR is 3.27). These results imply that this polymorphism may have functional significance in osteosarcoma carcinogenesis.

Osteosarcoma is one of major cancer types in the Guangxi Zhuang Autonomous Region; the possible risk factors of which include radiation exposure, foreign bodies, genetic predisposition, and so on. Increasing epidemiological evidence has shown that an individual susceptibility related to genetic factors might be associated with osteosarcoma carcinogenesis [5, 6].

While ARHGAP35 spans 87 kb on chromosome 19q13.3 and contains 7 exons and 6 introns (PubMed Databases). Its encoding protein is a 190 kDa protein consisting of three major functional domains: (1) an NH_2_-terminal GTP-binding domain (GBD), (2) a middle domain (MD), and (3) a COOH-terminal GAP domain, which displays specificity for GTP-bound RhoA [9, 26]. Functionally, ARHGAP35 plays important roles in promoting cell spreading, membrane protrusion, and cell polarity [15, 27]. Recently, several reports have shown that ARHGAP35 plays an important role in cancer formation and metastasis [19, 20, 28]. In 2008, Shen et al. [19] investigated the role of ARHGAP35 in the breast tumor kinase (Brk) signal pathway and found that it is a Brk substrate both in vitro and in vivo. Through this signal pathway, ARHGAP35 is phosphorylated at the Y1105 residue by Brk and next associated with p120RasGAP. As a consequence, ARHGAP35 is stimulated and p120 functions are attenuated, leading to RhoA inactivation and Ras activation, respectively. Their results show ARHGAP35 activation promotes breast cancer growth, migration, and invasion, and provide important evidence for the crucial roles of this Brk-ARHGAP35 signaling pathway in promoting breast malignancy [19]. In accordance with these reports, our present study exhibited that ARHGAP35 might be involved in osteosarcoma tumorigenesis.

With the Human Genome Project developing, more than one hundred polymorphisms have been identified in ARHGAP35 (dbSNP in NCBI Database). In this study, we only analyzed ARHGAP35 rs1052667 polymorphism, primarily because this polymorphism is relatively common in most populations, whereas other polymorphisms are rare. In this study, we collected 247 osteosarcoma and 428 control samples from Guangxi Zhuang Autonomous Region, a relatively high incident area of osteosarcoma. we found that about 20 percent of control individuals had ARHGAP35 rs1052667 T alleles, similar to the data from the Human Genome Project (dbSNP Database, web: http://www.ncbi.nlm.nih.gov/SNP/snp_ref.cgi?rs=rs1052667). However, higher frequency was observed in the individuals with osteosarcoma, and following analysis showed this polymorphism increased osteosarcoma risk. These results suggested ARHGAP35 rs1052667 polymorphism might modify the risk of tumors such as osteosarcoma.

This risk role might be related to the posttranscriptional regulation of gene expression. Because rs1052667 polymorphism locates at the 3′-UTR of ARHGAP35 gene, this variant might be involved in the regulation of mRNA stability and the control of mRNA subcellular localization [29]. Consequently, it may be associated with the functional dysregulation of ARHGAP35 and play a role in the carcinogenesis. Supporting the aforementioned hypothesis, recent studies have shown that the dysregulation of ARHGAP35 expression and function is involved in the tumorigenesis of some tumors such as lung cancer [30], melanoma [31], and breast cancer [19, 28, 32]. Thus, ARHGAP35 polymorphism might play an important role in the tumorigenesis of osteosarcoma, and this provided a new genetic insight into osteosarcoma tumorigenesis.

Additionally, we also investigated the association between ARHGAP35 rs1052667 polymorphism and osteosarcoma prognosis. We found that osteosarcoma patients having genotypes with ARHGAP35 rs1052667 T alleles had a significant poor RFS and OS compared to those without T alleles. Considering the difference of the treatment and to explore whether this difference affected the modifying role of ARHGAP35 rs1052667 polymorphism, we stratified the analysis of the effects of ARHGAP35 genotypes on osteosarcoma outcome by the treatment status. Results showed that this polymorphism modulated osteosarcoma prognosis, regardless of the radical or nonradical treatment status. Multivariate cox regression analysis next showed this polymorphism increased 1.53-times tumor reoccurrence risk and 0.91-times death risk; moreover, this risk did not depend on the clinical pathological change. This is possibly because it correlates with the fact that this polymorphism modifies tumor grade and differentiation and, consequently, might promote tumor proliferation and metastasis. Supporting our results, recent studies have exhibited that the dysregulation of ARHGAP35 promotes tumor growth, infiltration, and metastases and subsequently might result in poor prognosis of tumors [19, 20, 28, 30]. These data implied that ARHGAP35 rs1052667 polymorphism should be able to modify the prognosis of osteosarcoma and should be an important prognostic marker for this tumor.

In the present study, to control the effects of confounders such as age, gender, and race, we used an individually matched design. In the stratified analysis, no interactive effects were found, suggesting that these factors do not modify the correlation between ARHGAP35 rs1052667 polymorphism and osteosarcoma risk.

However, there were several limitations to our study. Potential selection bias might have occurred because the selection of control subjects in our study was hospital-based. Despite the analysis of ARHGAP35 rs1052667 polymorphism, we did not analyze other polymorphisms of this gene possibly able to modify the risk of osteosarcoma. Although this study is molecular epidemiological investigation based on clinic samples of osteosarcomas, it is deficient in functional analysis. Additionally, our findings were based on relatively small numbers and limited by small number of subjects in part of the genotype strata. Therefore, more genes deserve further elucidation based on a large sample and the combination of genes.

## 5. Conclusions

In summary, to the best of our knowledge, this is the first report investigating an association between ARHGAP35 rs1052667 polymorphism and osteosarcoma risk and prognosis in Guangxi patients. We have found evidence that the genotypes of ARHGAP35 rs1052667 T alleles may be correlated with increased risk and poor prognosis for osteosarcoma and that this polymorphism may be involved in the tumorigenesis of this type of tumor. Given that osteosarcoma is a highly fatal tumor, the finding of a genetic susceptibility (if confirmed) may have implications for screening and prevention.

## Figures and Tables

**Table 1:** Demographic and etiologic characteristics of osteosarcoma cases and controls.

Variable	Controls (*n* = 428)		Cases (*n* = 247)		*P*
	*n*	%	*n*		
Sex					
Male	265	61.9	154	62.3	0.934
Female	163	38.1	93	37.7	
Age (yrs)					
≤26	278	65.0	161	65.2	0.952
>26	150	35.0	86	34.8	
Race					
Han	279	65.2	159	64.4	0.555
Minority	149	34.8	88	35.6	
Smoking status					
No	398	93.0	231	93.5	0.792
Yes	30	7.0	16	6.5	
Drinking status					
No	402	93.9	233	94.3	0.829
Yes	26	6.1	14	5.7	
Paget's disease					
No	428	100.0	246	96.6	0.366
Yes	0	0.0	1	0.4	
Trauma					
No	411	96.0	228	92.3	0.038
Yes	17	4.0	19	7.7	
Radiation exposure					
No	415	97.0	236	95.5	0.339
Yes	13	3.0	11	4.5	
PBBL^b^					
No	428	100.0	246	96.6	0.366
Yes	0	0.0	1	0.4	

^a^The mean ± S.D. ages were 26.38 ± 15.51 and 26.31 ± 14.16 for cases and controls, respectively.

^b^PBBL refers to the preexisting benign bone lesions, including fibrous dysplasia, osteochondromatosis, and chondromatosis.

**Table 2:** The rs1052667 polymorphism of ARHGAP35 and osteosarcoma risk.

rs1052667	Controls		Cases		OR	*P*
	*n*	%	*n*	%		
Genotype						
CC	341	79.7	133	53.8	1	
CT	72	16.8	70	28.3	2.41 (1.64–3.55)^a^	9.00 × 10^−6^
TT	15	3.5	44	17.8	7.35 (3.95–13.68)^a^	3.12 × 10^−10^
CT/TT^b^	87	20.3	114	46.2	3.27 (2.31–4.61)^a^	1.92 × 10^−11^
Allele						
C	754	88.1	336	68.0	1	
T	102	11.9	158	32.0	2.25 (1.64–3.09)	4.43 × 10^−7^

^a^OR conditional on matched set adjusted by smoking and drinking status, radiation exposure history, trauma history, paget's disease history, and benign bone lesions.

^b^CT/TT represented the combination of rs1052667-CT genotype and rs1052667-TT genotype.

**Table 3:** The rs1052667 polymorphism of ARHGAP35 and osteosarcoma risk stratified by race (Han and minority), gender (female and male), and age (≤26 yrs and >26 yrs).

Variable	Genotype	Control		Case		OR (95% CI)^a^	*P*
		*n*	%	*n*	%		
Race^b^	rs1052667						
Han	CC	218	78.1	82	51.6	1	
	CT/TT	61	21.9	77	48.4	3.40 (2.22–5.19)	1.61 × 10^−8^
Minority	CC	123	82.6	51	58.0	1	
	CT/TT	26	17.4	37	42.0	3.39 (1.86–6.18)	6.67 × 10^−5^
Gender^c^	rs1052667						
Female	CC	129	79.1	49	52.7	1	
	CT/TT	34	20.9	44	47.3	3.42 (1.96–5.98)	1.53 × 10^−5^
Male	CC	212	80.0	84	54.5	1	
	CT/TT	53	20.0	70	45.5	3.29 (2.12–5.12)	1.08 × 10^−7^
Age^d^	rs1052667						
≤26	CC	214	77.0	80	49.7	1	
	CT/TT	64	23.0	81	50.3	3.40 (2.23–5.17)	1.11 × 10^−8^
>26	CC	127	84.7	53	61.6	1	
	CT/TT	23	15.3	33	38.4	3.35 (1.79–6.25)	1.50 × 10^−4^

^a^OR conditional on matched set.

^b^Likelihood ratio test for interaction of the stratified variable (Han and Minority) and rs1052667 genotype was calculated as test for the heterogeneity of ORs across strata (interact term OR = 1.02, *P*_interaction_ = 0.957).

^c^Likelihood ratio test for interaction of the stratified variable (male and female) and rs1052667 genotype was calculated as test for the heterogeneity of ORs across strata (interact term OR = 0.99, *P*_interaction_ = 0.983).

^d^Likelihood ratio test for interaction of the stratified variable (age: ≤26 yrs and >26 yrs) and rs1052667 genotype was calculated as test for the heterogeneity of ORs across strata (interact term OR = 1.01, *P*_interaction_ = 0.982).

**Table 4:** The rs1052667 polymorphism of ARHGAP35 and the prognosis of osteosarcoma.

Rs1052667	Overall survival		Recurrence-free survival	
Genotype	HR (95% CI)	*P*	HR (95% CI)	*P*
CC	1		1	
CT	1.57 (1.16–2.12)	3.86 × 10^−3^	1.82 (1.33–2.50)	2.12 × 10^−4^
TT	1.91 (1.32–2.77)	5.95 × 10^−4^	2.53 (1.73–3.70)	1.73 × 10^−6^

**Table 5:** The rs1052667 polymorphism of ARHGAP35 and clinic pathological features of osteosarcoma.

Variable	rs1052667-CC		rs1052667-CT/TT		OS (95% CI)	*P*
	*n*	%	*n*	%		
Age (yrs)						
≤26	80	60.2	81	71.1	1	
>26	53	39.8	33	28.9	0.72 (0.39–1.35)	0.31
Gender						
Female	49	36.8	44	38.6	1	
Male	84	63.2	70	61.4	1.07 (0.61–1.89)	0.82
Race						
Han	82	61.7	77	67.5	1	
Minority	51	38.3	37	32.5	0.98 (0.54–1.77)	0.95
Tumor site						
Femur	72	54.1	68	59.6	1	
Tibia	30	22.6	21	18.4	0.66 (0.32–1.33)	0.24
Humeral bone	22	16.5	16	14	0.90 (0.40–2.01)	0.79
Others	9	6.8	9	7.9	0.91 (0.31–2.70)	0.86
Tumor size						
≤5 cm	57	42.9	15	13.2	1	
>5 cm	76	57.1	99	86.8	4.85 (2.51–9.37)	2.69 × 10^−6^
Tumor grade						
Low	68	51.1	23	20.2	1	
High	65	48.9	91	79.8	4.07 (2.28–7.37)	2.14 × 10^−6^
Metastasis						
No	87	65.4	45	39.5	1	
Yes	46	34.6	69	60.2	2.78 (1.63–4.76)	1.88 × 10^−4^
